# Supplementary material for: Single voxel autocorrelation reflects hippocampal function in temporal lobe epilepsy
Source: Imaging Neurosci (Camb). 2026 Jan 22;4:IMAG.a.1108. doi: 10.1162/IMAG.a.1108 (PMC12828354; doi:10.1162/IMAG.a.1108)
Supplement: Supplementary Material [file IMAG.a.1108_supp.pdf]

Supplemental materials

Single voxel autocorrelation reflects hippocampal function in temporal lobe epilepsy

Nichole R. Bouffard, Sam Audrain, Ali M. Golestani, Morgan D. Barense, Morris Moscovitch & Mary Pat McAndrews

Corresponding author: Nichole R. Bouffard

Email: bouffard@wustl.edu

Supplemental Materials: Table of Contents

SECTION/ITEM	DESCRIPTION	PAGE
TABLE S1	**Clinical characteristics of each individual patient**	**3-4**
ANALYSIS	**Reliability analysis – Overlap of individual autocorrelation clusters with group average (within-group)**	**5-6**
FIGURE S1	**Cluster Replication Validation: Spatial permutation of clusters relative to prior work**	**7**
FIGURE S2	**Autocorrelation cluster reliability – Spatial overlap of autocorrelation clusters between individuals within the same group**	**8**
TABLE S2	**Size of autocorrelation clusters (average number of voxels per cluster)**	**9**
TABLE S3	**Average single voxel autocorrelation per cluster**	**10**
TABLE S4	**Correlation between average autocorrelation and memory performance**	**11**
TABLE S5	**Cluster preservation (Jaccard coefficients) for each autocorrelation cluster**	**12**
FIGURE S3	**Correlations with change in verbal/visuospatial memory scores**	**13**
FIGURE S4	**Cluster Preservation Validation: Spatial permutation of cluster preservation**	**14**
ANALYSIS	**Voxel-level analysis of single voxel autocorrelation**	**15-16**
ANALYSIS	**Relationship between autocorrelation and disease duration**	**17**
ANALYSIS	**Flipped hemisphere analysis**	**18-19**
FIGURE S5	**Spatial distribution of the autocorrelation clusters along the anterior-posterior axis**	**20**
FIGURE S6	**Spatial distribution of the autocorrelation clusters along the medial-lateral axis**	**21**

Table S1. Clinical characteristics of each individual patient

**ID**	**Laterality (Left or Right TLE)**	**Age**	**Sex**	**Age of seizure onset**	**Disease duration (years)**	**Latest Engel**	**MRI findings**
1	LTLE	48	F	16	32	1a	normal *
2	LTLE	52	F	38	14	1a	LMTS *
3	LTLE	24	F	<1	23.33	NA	LMTS
4	LTLE	43	F	<1	42.25	NA	LMTS
5	LTLE	38	F	30	8	1c	normal *
6	LTLE	35	F	33	2	1c	LMTS
7	LTLE	35	F	13	22	1d	normal *
8	LTLE	24	F	1.5	22.5	1b	LMTS *
9	RTLE	38	F	18	20	1a	RMTL Tumor *
10	RTLE	26	F	23	3	1a	normal
11	RTLE	57	M	44	13	NA	normal
12	RTLE	36	F	30	6	1d	RMTS *
13	RTLE	19	M	17	2	NA	RAGNH
14	RTLE	34	F	27	7	NA	RMTS
15	RTLE	44	F	7	37	1a	RMTS *
16	RTLE	18	M	17	1	1a	RMTL Ganglioglioma *
17	RTLE	56	M	37	19	1a	RMTS *
18	LTLE	35	F	2	33	1c	LMTS
19	LTLE	53	F	7	46	1a	LMTS *
20	LTLE	42	F	7	35	NA	LMTS *
21	RTLE	43	M	5	38	1d	RMTS *
22	RTLE	23	M	18	5	1a	RMTS *
23	RTLE	34	M	23	11	1a	RMTS *
24	RTLE	22	M	16	6	1a	LMTS
25	RTLE	48	M	<1	48	1a	RMTS *
26	RTLE	48	F	23	25	NA	generalized atrophy
27	RTLE	50	F	42	8	1d	RMTS *
28	RTLE	53	M	28	25	NA	RMTS
29	RTLE	25	M	15	10	NA	BHippMal + RAGNH
30	RTLE	25	M	21	4	1a	RMTS *
31	RTLE	24	M	12	12	1a	RMTS *
32	RTLE	58	M	51	7	NA	RMTS **
33	RTLE	50	M	9	41	NA	normal
34	LTLE	33	M	26	7	1a	normal *
35	LTLE	44	M	38	6	1a	LMTS

36	LTLE	25	M	2	23	1a	LMTS *
37	LTLE	57	M	<1	56.5	1a	LMTS *
38	LTLE	31	F	26	5	2a	LMTS *
39	LTLE	23	M	20	3	1a	LMTS *
40	LTLE	26	M	1	25	1a	LMTS *
41	LTLE	58	M	48	10	1a	LMTL DNET *
42	RTLE	58	F	16	42	1a	RMTS *
43	RTLE	32	F	28	4	1a	RMTL CtxDysp **
44	RTLE	24	M	4	20	1a	RMTS *
45	RTLE	18	M	12	6	1d	RMTS *
46	LTLE	25	F	20	5	1a	normal *
47	LTLE	18	F	<1	17.2	1b	LMTS
48	LTLE	59	F	44	15	1a	normal *
49	LTLE	33	M	21	12	1d	LAGNH **

* MRI findings confirmed by pathology; ** MRI findings not confirmed by pathology; CtxDysp=cortical dysplasia; DNET=dysembryoplastic neuroepithelial tumor; F=female; L=left; LAGNH=left amygdala glioneuronal hamartoma; LMTL=left medial temporal lobe; LMTS= left medial temporal sclerosis; RMTS=right medial temporal sclerosis; RMTL=right medial temporal lobe; RAGNH=right amygdala glioneuronal hamartoma;

Reliability analysis – Overlap of individual autocorrelation clusters with group average (within-group)

We examine how reliable the spatial overlap of autocorrelation clusters was within each group. For example, for a patient with LTLE, how similar was the spatial organization of their autocorrelation clusters to all other LTLE patients. The aims of this analysis were to 1. Determine how consistent the spatial organization of autocorrelation clusters was among individuals in the same group and 2. Whether there was more variability in spatial organization of autocorrelation clusters among individuals in the LTLE and RTLE groups compared to Controls. To measure spatial overlap of autocorrelation clusters, we calculated the Jaccard coefficient between each individual's cluster map with every other individual's cluster map in their respective group (Controls, LTLE and RTLE). High spatial overlap (i.e., large Jaccard coefficients) between clusters of individuals from the same group suggests a high reliability of spatial organization of autocorrelation clusters across individuals within groups. Low spatial overlap (i.e., small Jaccard coefficients) between clusters of individuals from the same group suggests low reliability of spatial organization of autocorrelation clusters across individuals within groups. After computing the within-group Jaccard coefficients, we ran a linear mixed effects model on the within-group Jaccard coefficients with cluster (anterior-medial, intermediate, posterior-lateral), group (Controls, LTLE, RTLE), and hemisphere (left, right) as predictors.

We found a significant main effect of cluster ($F(2,355) = 6499.32$, $p < 0.001$), and a significant main effect of hemisphere ($F(1,355) = 142.50$, $p < 0.001$). We did not find a significant main effect of group ($p = 0.183$). We also found a significant interaction

between group and hemisphere ($F(2,355) = 7.60$, $p < 0.001$) and a significant interaction between cluster and hemisphere ($F(2,355) = 27.01$, $p < 0.001$).

Post hoc analysis of the main effect of cluster revealed that the anterior-medial and posterior-lateral clusters had greater overlap (higher Jaccard coefficients) compared to the intermediate cluster (anterior-medial > intermediate: $t(355)=89.48$, $p < 0.001$; posterior-lateral > intermediate: $t(355)=105.92$, $p < 0.001$), suggesting greater reliability of the anterior-medial cluster and posterior-lateral cluster and lower reliability of the intermediate cluster. This finding is consistent with Bouffard, Golestani, et al. (2022) and Coughlan et al. (2022), where the intermediate cluster had low reliability and was found to be inconsistent across individuals and across runs from the same individual. We also found that the posterior-lateral cluster had higher Jaccard coefficients than the anterior-medial cluster ($t(355) = 16.43$, $p < 0.001$). These results address the first aim of our analysis and conclude that the anterior-medial and posterior-lateral clusters are the most reliable clusters. Post hoc analysis of the main effect of hemisphere revealed that the right hemisphere had greater Jaccard coefficients than the left hemisphere ($t(355) = 11.93$, $p < 0.001$). This suggests that the spatial organization of clusters in the right hemisphere were more reliable among individuals of the same group than the left hemisphere.

Notably we did not find a main effect of group. This result addresses the second aim of our analysis and suggests that the variability in spatial organization of autocorrelation clusters among individuals in the Control group was not significantly different from the individuals in the LTLE and RTLE groups.

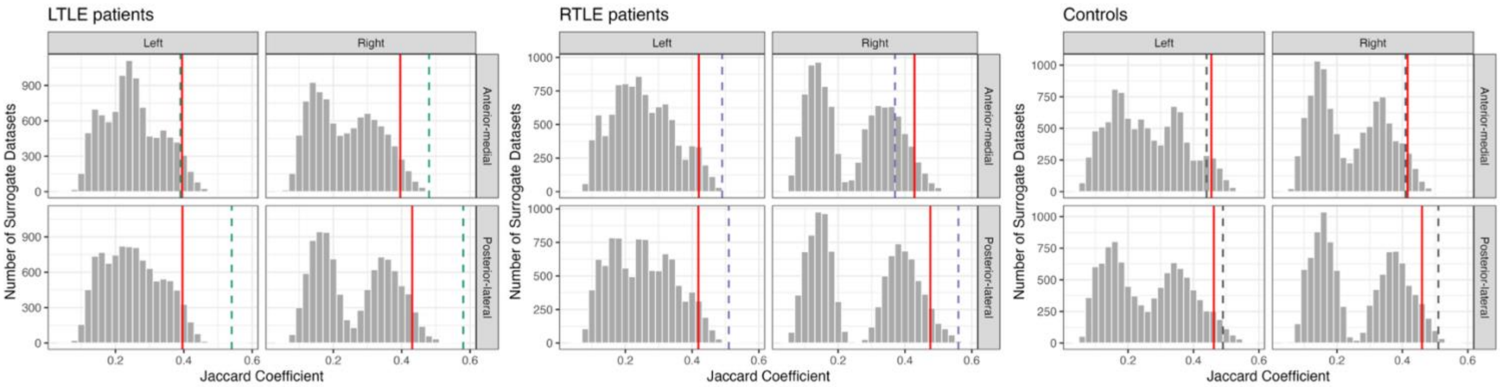

Figure S1. Cluster Replication Validation: Spatial permutation of clusters relative to prior work. Data from the group-level LTLE, RTLE, and healthy Control participants was used to generate surrogate datasets (10,000 surrogate datasets per group). Each surrogate map generated preserved the spatial autocorrelation of the fMRI data while removing spatial specificity (method adapted from Burt et al., 2020). Autocorrelation clusters were generated for each surrogate dataset and the Jaccard coefficient was computed between each surrogate cluster map and the cluster map from our previous study (Bouffard, Golestani, et al., *Cerebral Cortex*, 2023). The resulting null distribution of Jaccard coefficients was generated for each group (LTLE, RTLE, Control), each cluster (anterior-medial and posterior-lateral) and each hemisphere (left/right). The cluster preservation values for the actual data are denoted with dashed lines (LTLE = green, RTLE = purple, Controls = grey). In the LTLE and RTLE patients, autocorrelation clusters were replicated and had high overlap with our prior work in both hemispheres, except for the anterior-medial cluster of the epileptogenic hemisphere, which had values below the 95th percentile. In Controls, we found a replication of the posterior-lateral cluster in both hemispheres, however the anterior-medial cluster did not exceed the 95th percentile in either hemisphere.

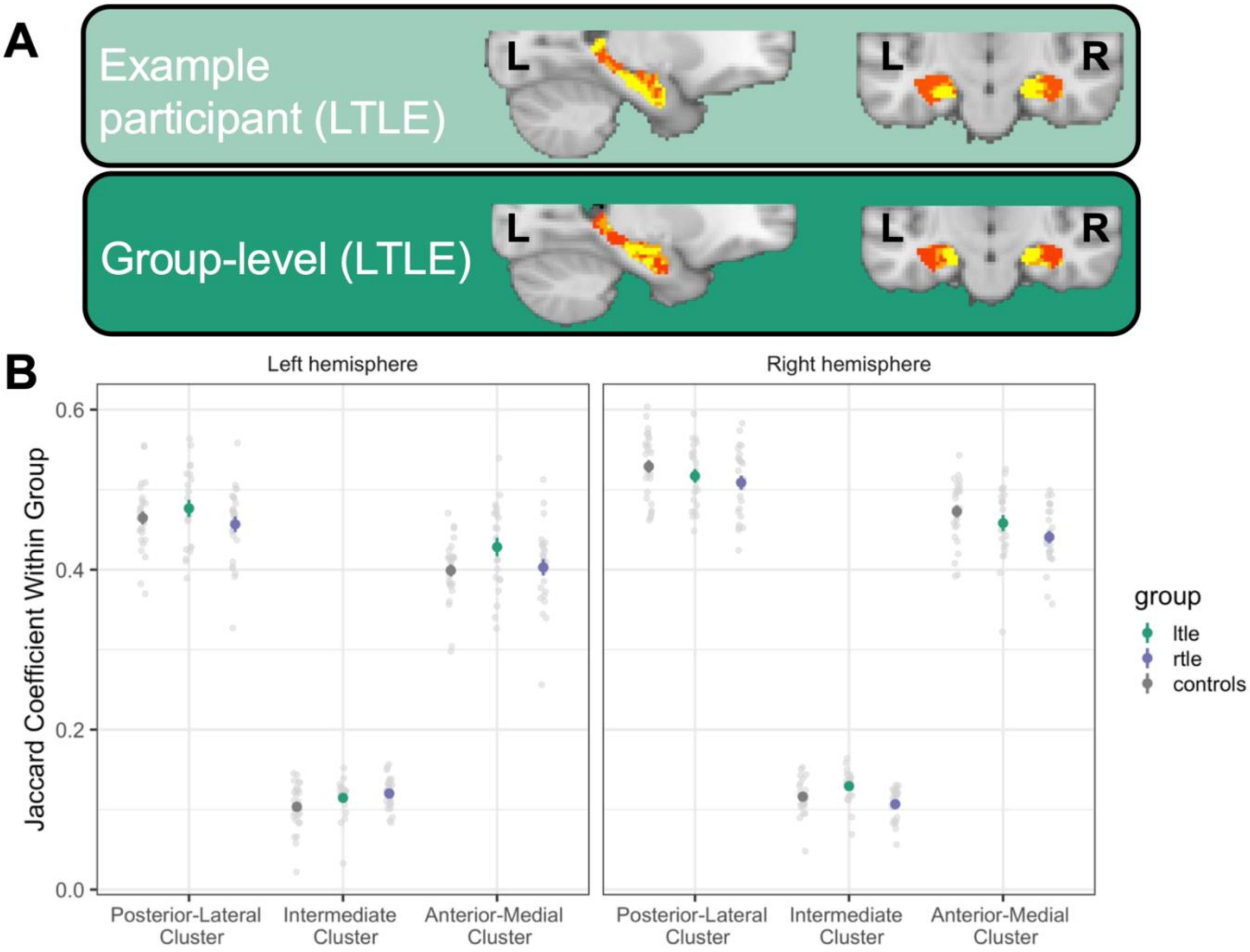

Figure S2. Autocorrelation cluster reliability – Spatial overlap of autocorrelation clusters between individuals within the same group. (A) An example autocorrelation cluster map from one LTLE individual (top) and the group-level autocorrelation cluster map from the LTLE group (bottom) **(B)** The Jaccard coefficient between each individual's cluster map with every other individual's cluster map in their respective group (Controls, LTLE and RTLE). High spatial overlap (i.e., large Jaccard coefficients) between clusters of individuals from the same group suggests reliable spatial organization of autocorrelation clusters across individuals within groups.

Table S2. Size of autocorrelation clusters (average number of voxels per cluster)

Group	Hemisphere	MTS status	Cluster	Average (SD)
BNA Whole Hipp mask	L	—	—	1161
BNA Whole Hipp mask	R	—	—	1098
Controls	L	noMTS	Posterior-lateral	557.11 (37.46)
Controls	L	noMTS	Intermediate	207.82 (73.07)
Controls	L	noMTS	Anterior-medial	396.07 (64.97)
Controls	R	noMTS	Posterior-lateral	539.54 (31.81)
Controls	R	noMTS	Intermediate	187.36 (45.05)
Controls	R	noMTS	Anterior-medial	371.11 (34.26)
LTLE	L	MTS	Posterior-lateral	560.53 (50.24)
LTLE	L	MTS	Intermediate	195.67 (56.5)
LTLE	L	MTS	Anterior-medial	404.8 (37.62)
LTLE	L	noMTS	Posterior-lateral	544.14 (10.73)
LTLE	L	noMTS	Intermediate	254.57 (57.24)
LTLE	L	noMTS	Anterior-medial	362.29 (64.42)
LTLE	R	MTS	Posterior-lateral	515.07 (38.1)
LTLE	R	MTS	Intermediate	223.07 (42.41)
LTLE	R	MTS	Anterior-medial	359.87 (35.94)
LTLE	R	noMTS	Posterior-lateral	530.86 (31.98)
LTLE	R	noMTS	Intermediate	222.86 (38.22)
LTLE	R	noMTS	Anterior-medial	344.29 (13.51)
RTLE	L	MTS	Posterior-lateral	542.59 (31.34)
RTLE	L	MTS	Intermediate	220.35 (44.04)
RTLE	L	MTS	Anterior-medial	398.06 (36.06)
RTLE	L	noMTS	Posterior-lateral	553.75 (27.21)
RTLE	L	noMTS	Intermediate	210.25 (70.38)
RTLE	L	noMTS	Anterior-medial	397 (65.07)
RTLE	R	MTS	Posterior-lateral	526.47 (37.17)
RTLE	R	MTS	Intermediate	171.88 (49.04)
RTLE	R	MTS	Anterior-medial	399.65 (47.82)
RTLE	R	noMTS	Posterior-lateral	528 (50.74)
RTLE	R	noMTS	Intermediate	170 (45.1)
RTLE	R	noMTS	Anterior-medial	400 (32.29)

Table S3. Average single voxel autocorrelation per cluster

Group	Hemisphere	MTS status	Cluster	Average Autocorrelation	SD
Controls	L	noMTS	Posterior-lateral	0.0507	0.0134
Controls	L	noMTS	Intermediate	0.0817	0.0249
Controls	L	noMTS	Anterior-medial	0.236	0.149
Controls	R	noMTS	Posterior-lateral	0.0527	0.0138
Controls	R	noMTS	Intermediate	0.0888	0.0259
Controls	R	noMTS	Anterior-medial	0.243	0.109
LTLE	L	MTS	Posterior-lateral	0.0783	0.0407
LTLE	L	MTS	Intermediate	0.13	0.0714
LTLE	L	MTS	Anterior-medial	0.365	0.208
LTLE	L	noMTS	Posterior-lateral	0.0575	0.0129
LTLE	L	noMTS	Intermediate	0.0896	0.0239
LTLE	L	noMTS	Anterior-medial	0.234	0.0981
LTLE	R	MTS	Posterior-lateral	0.0743	0.0377
LTLE	R	MTS	Intermediate	0.121	0.067
LTLE	R	MTS	Anterior-medial	0.349	0.208
LTLE	R	noMTS	Posterior-lateral	0.0604	0.0147
LTLE	R	noMTS	Intermediate	0.105	0.0319
LTLE	R	noMTS	Anterior-medial	0.291	0.12
RTLE	L	MTS	Posterior-lateral	0.0645	0.023
RTLE	L	MTS	Intermediate	0.0971	0.036
RTLE	L	MTS	Anterior-medial	0.219	0.0879
RTLE	L	noMTS	Posterior-lateral	0.0565	0.0201
RTLE	L	noMTS	Intermediate	0.0868	0.0334
RTLE	L	noMTS	Anterior-medial	0.185	0.0716
RTLE	R	MTS	Posterior-lateral	0.0621	0.0198
RTLE	R	MTS	Intermediate	0.0999	0.0351
RTLE	R	MTS	Anterior-medial	0.24	0.151
RTLE	R	noMTS	Posterior-lateral	0.0584	0.0146
RTLE	R	noMTS	Intermediate	0.0912	0.0238
RTLE	R	noMTS	Anterior-medial	0.22	0.0756

Table S4. Correlation between average autocorrelation and memory performance

Group	Hemisphere	Cluster	Verbal Memory (Pearson's R)	P value	P_{FDR} corrected
LTLE	Left	Posterior-lateral	-0.34	0.12	0.48
LTLE	Left	Anterior-medial	-0.10	0.65	1.0
LTLE	Right	Posterior-lateral	-0.35	0.11	0.44
LTLE	Right	Anterior-medial	-0.21	0.35	0.70
**Group**	**Hemisphere**	**Cluster**	**Visuospatial Memory (Pearson's R)**	**P value**	**P_{FDR} corrected**
RTLE	Left	Posterior-lateral	0.07	0.75	1.0
RTLE	Left	Anterior-medial	-0.16	0.45	1.0
RTLE	Right	Posterior-lateral	0.11	0.59	1.0
RTLE	Right	Anterior-medial	-0.13	0.53	1.0

Table S5. Cluster preservation (Jaccard coefficients) for each autocorrelation cluster

Group	Hemisphere	MTS status	Cluster	Jaccard coefficient	SD
LTLE	L	MTS	Posterior-lateral	0.495	0.0696
LTLE	L	MTS	Anterior-medial	0.428	0.107
LTLE	L	noMTS	Posterior-lateral	0.479	0.0451
LTLE	L	noMTS	Anterior-medial	0.418	0.0762
LTLE	R	MTS	Posterior-lateral	0.517	0.0433
LTLE	R	MTS	Anterior-medial	0.503	0.0717
LTLE	R	noMTS	Posterior-lateral	0.507	0.0426
LTLE	R	noMTS	Anterior-medial	0.482	0.0844
RTLE	L	MTS	Posterior-lateral	0.466	0.0603
RTLE	L	MTS	Anterior-medial	0.41	0.0978
RTLE	L	noMTS	Posterior-lateral	0.488	0.0232
RTLE	L	noMTS	Anterior-medial	0.426	0.101
RTLE	R	MTS	Posterior-lateral	0.493	0.0468
RTLE	R	MTS	Anterior-medial	0.472	0.0808
RTLE	R	noMTS	Posterior-lateral	0.516	0.0454
RTLE	R	noMTS	Anterior-medial	0.486	0.08

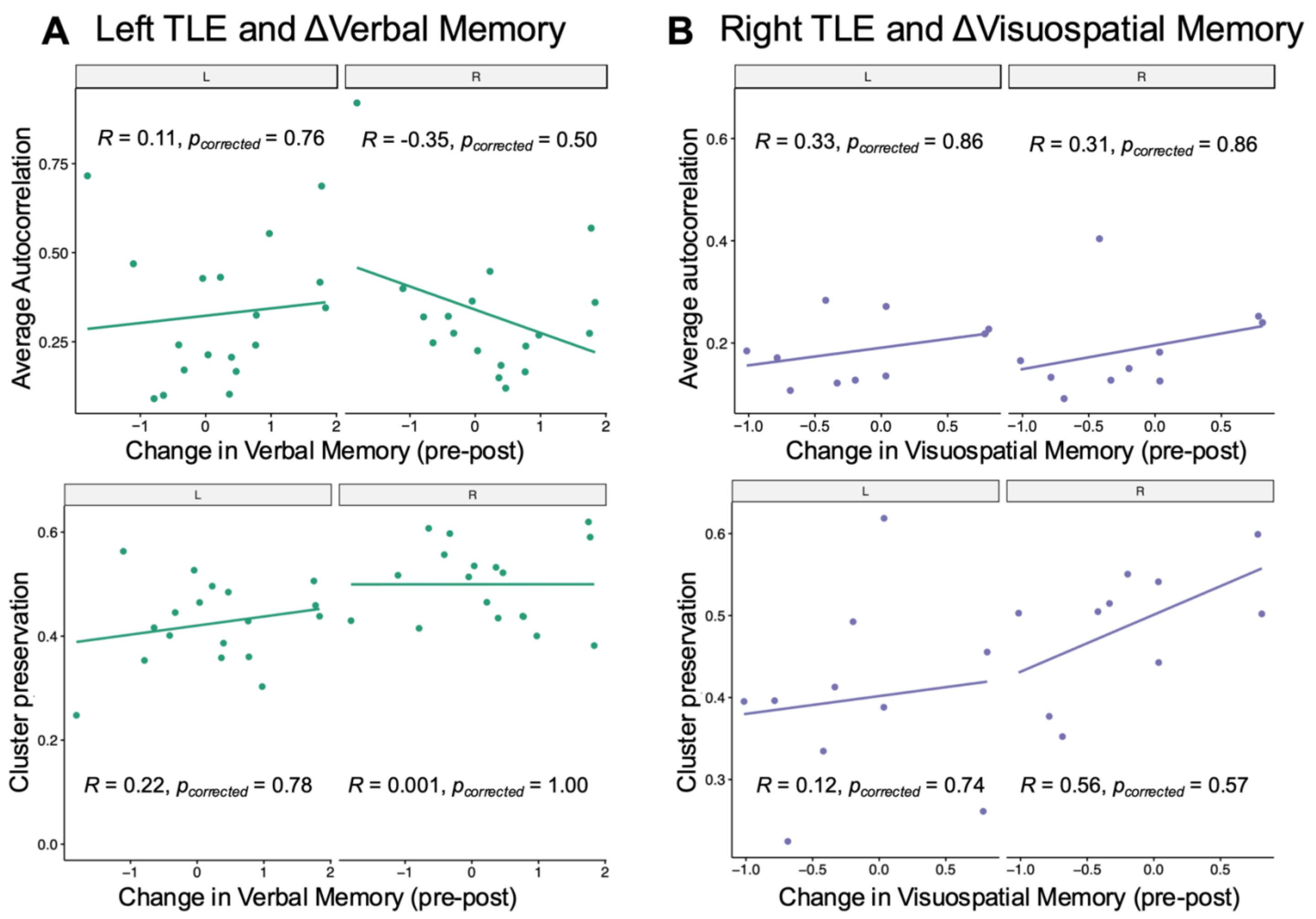

Figure S3. Correlations with change in verbal/visuospatial memory scores. We first computed the average change in verbal memory scores (pre- minus post-surgery) for LTLE (M = 0.23 (SD =0.98)) and RTLE (M = -0.44 (SD =0.70)) individuals who elected to get surgery (note, we had post-surgery neuropsychological scores from 20 LTLE and 11 RTLE patients). We also computed the average change in visuospatial memory scores (pre- minus post-surgery) for LTLE (M = 0.12 (SD =0.82)) and RTLE (M = -0.18 (SD =0.59)). **(A)** The Pearson's correlation between the change in verbal memory with the average autocorrelation (top) and cluster preservation (bottom) in anterior-medial cluster in the left and right hemisphere of LTLE patients. There were no significant correlations that survived multiple comparisons correction (FDR). **(B)** The Pearson's correlation between the change in visuospatial memory with the average autocorrelation (top) and cluster preservation (bottom) in anterior-medial cluster in the left and right hemisphere of RTLE patients. There were no significant correlations that survived multiple comparisons correction (FDR).

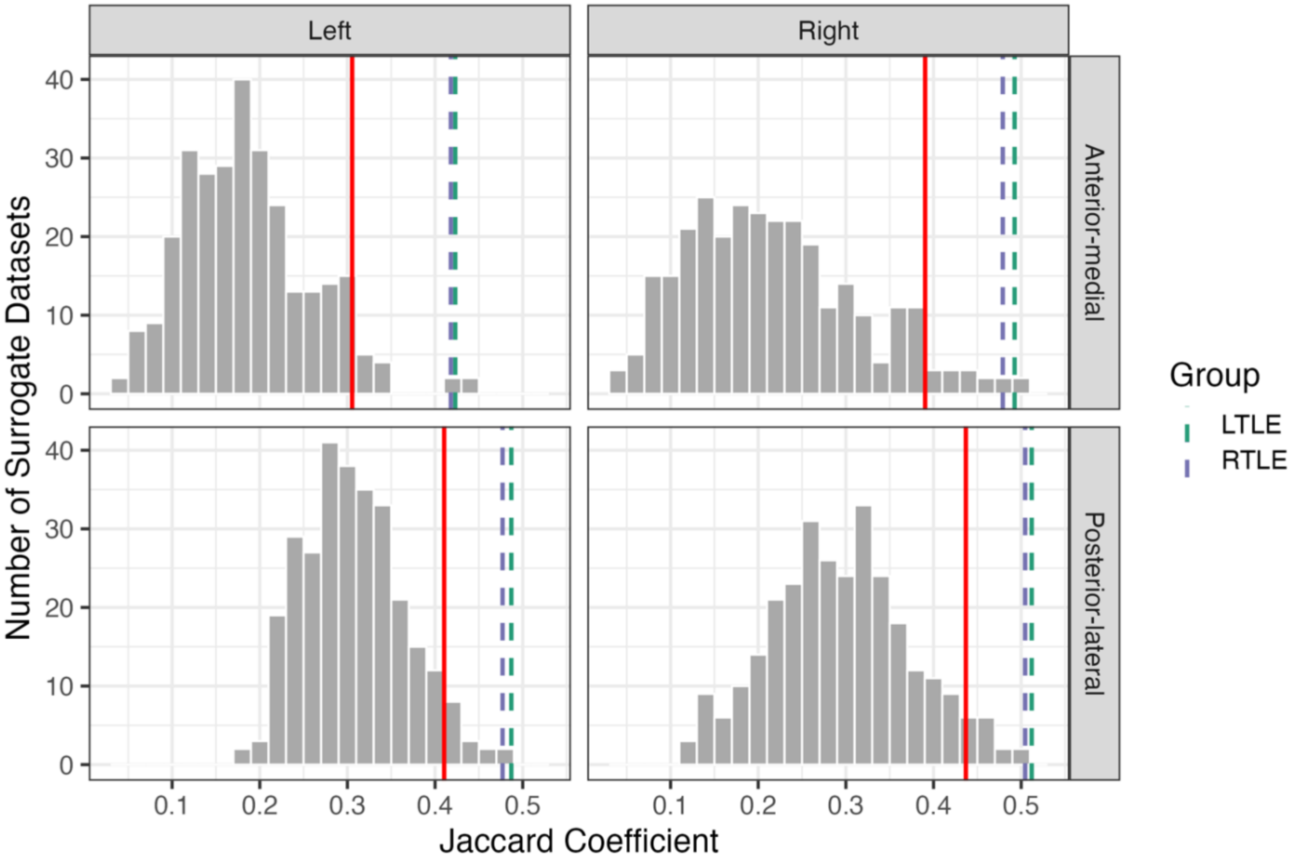

Figure S4. Cluster Preservation Validation: Spatial permutation of cluster preservation. Data from the healthy Control participants was used to generate surrogate datasets (10 surrogate datasets per participant). Each surrogate map generated preserved the spatial autocorrelation of the fMRI data while removing spatial specificity (method adapted from Burt et al., 2020). Autocorrelation clusters were generated for each surrogate dataset and the Jaccard coefficient between each individual surrogate cluster map and the original, group-level cluster map for Controls was computed. A null distribution of cluster preservation was generated for each cluster (anterior-medial and posterior-lateral) and each hemisphere (left/right). The 95th percentile of the null distributions are denoted with a solid red line. For the anterior-medial cluster, the 95th percentile was 0.30 (left hemisphere) and 0.39 (right hemisphere). For the posterior-lateral cluster it was 0.41 (left hemisphere) and 0.43 (right hemisphere). The actual cluster preservation values for patients are included in purple (RTLE) and green (LTLE) dashed lines. The cluster preservation of the original patient clusters (anterior-medial and posterior-lateral) exceeded the 95th percentile in both hemispheres.

Voxel-level analysis of single voxel autocorrelation

A voxel-voxel approach is a fine-grained approach to examining changes in autocorrelation in each individual voxel, without clustering the voxels. We were interested in whether this fine-grained approach might reveal a difference between controls and patients that was potentially obscured by our autocorrelation clusters. Therefore we ran a linear mixed effect model with the voxel-level autocorrelation as the dependent variable. Specifically, the dependent variable was the autocorrelation for every voxel in the hippocampus at lag 1. Fixed effect predictors included hemisphere (left, right) and group (controls (N = 28), LTLE (N = 23), RTLE (N = 26) and participant as the random intercept in the random effects term. We found a significant main effect of hemisphere ($F(1,173863) = 47.63$, $p <0.001$), a significant main effect of group ($F(2, 74) = 5.23$, $p <0.01$), and a significant hemisphere x group interaction ($F(2, 173863) = 6.86$, $p <0.001$). Note that the number of observations was too high to calculate the degrees of freedom, therefore for the following post-hoc tests an asymptotic method was used (with p's corrected for multiple comparisons using the Tukey method). Post hoc analysis of the main effect of hemisphere revealed that the right hemisphere had significantly higher autocorrelation than the left ($z(inf) = 6.90$, $p <0.001$). Post hoc analysis of the main effect of group revealed that LTLE had greater autocorrelation than controls ($z(inf) = 2.97$, $p <0.01$) and LTLE had greater autocorrelation than RTLE ($z(inf) = 2.67$, $p <0.05$). There was no difference between RTLE and controls ($z(inf) = 0.27$, $p= 0.96$). The post hoc analysis of the group x hemisphere interaction revealed the right hemisphere was greater than the left hemisphere in the control group ($z(inf) = 5.42$, $p < 0.001$) and the RTLE group ($z(inf) = 5.93$, $p < 0.001$). Also the LTLE group had higher autocorrelation in the left hemisphere compared to the left hemisphere of controls ($z(inf) = 3.15$, $p < 0.05$) and RTLE($z(inf) = 2.87$, $p < 0.05$). These findings are similar to what was reported in the original manuscript using the average autocorrelation per cluster. The one difference is that here the main effect of hemisphere was significant whereas before it wasn't.

Another approach would be to include voxel a random intercept. We also conducted the linear mixed effects model on the autocorrelation of each voxel and included hemisphere (left, right) and group (controls (N = 28), LTLE (N = 23), RTLE (N = 26) as

the fixed effect predictors and participant and voxel as the two different random intercepts in the random effects terms. We found very similar findings to the previous model, where there was a significant main effect of hemisphere ($F(1,173680.70) = 50.75$, $p < 0.001$), a significant main effect of group ($F(2, 74) = 5.23$, $p < 0.01$), and a significant hemisphere x group interaction ($F(2, 173202.89) = 7.22$, $p < 0.001$).

Although our original cluster method was the best approach to analyzing the group differences between controls, LTLE and RTLE patients, we were curious whether the voxel-to-voxel approach might be particularly sensitive to differences in MTS. To test this we ran linear mixed effects model on the autocorrelation (lag 1) for every voxel in the hippocampus and included hemisphere (Left, Right), group (LTLE, RTLE), and MTS (noMTS, MTS) as the fixed effect predictors. Note, there were fifteen LTLE patients with MTS and sixteen RTLE patients with MTS. We included participant as the random intercept in the random effects term. We found a significant effect of hemisphere ($F(1, 106122) = 48.72$, $p < 0.001$) but the main effect of group and MTS were not significant. We also found a significant interaction between hemisphere and MTS ($F(1, 106122) = 50.38$, $p < 0.001$). Post hoc analysis of the main effect of hemisphere revealed that the right hemisphere had significantly higher autocorrelation than the left ($z(inf) = 6.98$, $p < 0.0001$). Post hoc analysis of the interaction between hemisphere and MTS revealed that the right hemisphere had greater autocorrelation than the left in patients with no MTS ($z(inf) = 8.53$, $p < 0.001$). No other comparisons survived multiple comparison corrections. Again, here even with a fine-grained voxel-level approach, we still were not able to find robust differences in autocorrelation due to MTS.

Relationship between autocorrelation and disease duration

To investigate whether there is a relationship between autocorrelation and disease duration, we ran a linear mixed effects model on the average autocorrelation with cluster (anterior-medial, intermediate, posterior-lateral), group (LTLE (N=23), RTLE (N=26)), and hemisphere (left, right), and disease duration (z-scored) as a fixed effect predictors. We included participant as the random intercept in the random effects term. We did not find a main effect or any interactions with disease duration. We found a significant effect of cluster ($F(2,225) = 233.79$, $p < 0.001$), group ($F(1,45) = 5.84$, $p < 0.05$) and a cluster x group interaction ($F(2,225) = 16.01$, $p < 0.001$). These main effects and interaction were similar to the results reported in the original manuscript. Because our original findings still held when we included disease duration in the model, this suggests that disease duration does not explain the variance that is being captured by cluster and group.

Flipped hemisphere analysis

To increase statistical power and potentially increase our ability to detect differences between patients with and without MTS, we conducted a flipped hemisphere analysis where we normalized both groups to the control group and then used a grouping variable of “epileptogenic hemisphere” and “healthy hemisphere” instead of left and right hemisphere. We first used the average autocorrelation from the control group to mean center the autocorrelation values for each cluster in the LTLE and RTLE patient groups. After subtracting the average autocorrelation of control clusters from the patient clusters, we recoded the hemisphere variable. The new variable was a binary variables with two values: epileptogenic or healthy. For example, values from LTLE patients that had MTS in their left hemisphere were coded as epileptogenic whereas values from the right hemisphere were coded as “healthy.” We excluded participants that had possible abnormalities but were not confirmed with MTS. This resulted in LTLE (N = 22, 15 with MTS) and RTLE (N = 25, 17 with MTS). We investigated whether using this new hemisphere variable boosted our statistical power and resulted in different results compared to our previous approach where we used hemisphere (Left, Right) and MTS (MTS, no MTS) in our models. We applied this new approach to investigate the average autocorrelation and the cluster preservation.

We first ran a linear mixed effects model on the average autocorrelation (mean-centered with Controls) with cluster (anterior-medial, intermediate, posterior-lateral), group (LTLE, RTLE) and the new hemisphere variable (epileptogenic, healthy) as the fixed effect predictors. Participant was the random intercept in the random effects term. We found a significant effect of group ($F(1,46.98) = 5.04$, $p < 0.05$) and a significant cluster x group interaction ($F(2,225.02) = 11.45$, $p < 0.001$). There was a trending effect of cluster ($F(2,225.02) = 2.67$, $p = 0.07$). Importantly there was no significant effect of the new hemisphere variable ($F(1,247.57) = 0.96$, $p = 0.32$).

We next ran a linear mixed effects model on cluster preservation. Note the cluster preservation is measure that is already corrected for with controls (since it is the overlap between patient and control clusters), we did not need to do any additional manipulations. The model included cluster (anterior-medial, intermediate, posterior-

lateral), group (LTLE, RTLE) and the new hemisphere variable (epileptogenic, healthy) as the fixed effect predictors. Participant was the random intercept in the random effects term. We found a significant effect of cluster ($F(2,224.99) = 1270.01$, $p < 0.001$), a significant interaction between group and the new hemisphere variable ($F(1,256.80) = 17.64$, $p < 0.001$), and a significant three-way interaction between cluster x group x and the new hemisphere variable ($F(2,224.99) = 4.76$, $p < 0.01$). There was not a significant effect of the new hemisphere variable ($F(1,256.80) = 2.83$, $p = 0.094$). We performed a post hoc analysis of the group x new hemisphere interaction and found that the healthy hemisphere in LTLE patients had higher cluster preservation than the epileptogenic hemisphere in LTLE patients ($t(256.8) = 4.019$, $p < 0.001$).

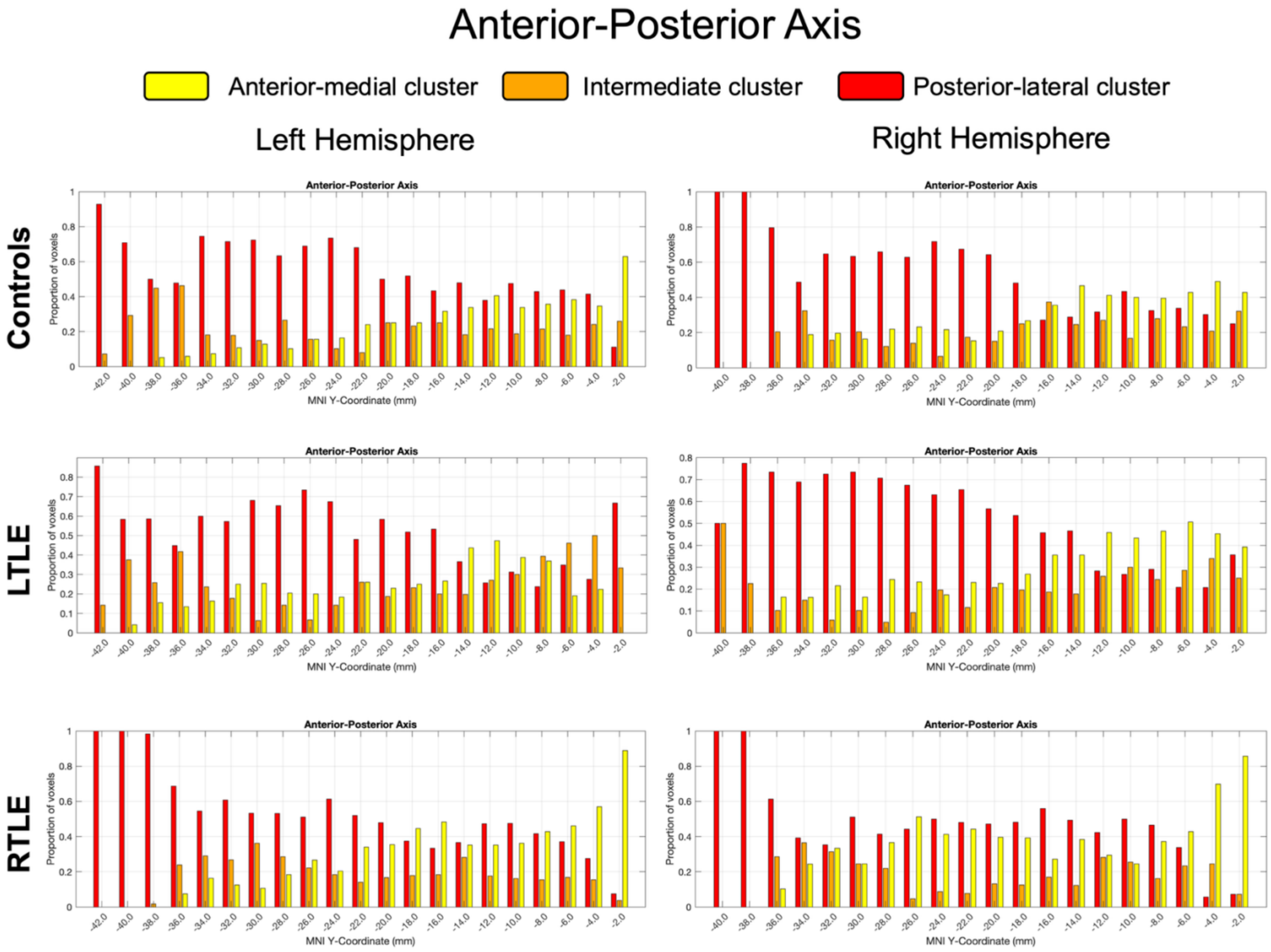

Figure S5. Spatial distribution of the autocorrelation clusters along the anterior-posterior axis. The proportion of voxels assigned to the anterior-medial cluster, intermediate cluster, and posterior-lateral cluster across 2 mm slices in MNI space along the anterior-posterior axis (Y direction). The spatial distribution of autocorrelation clusters is plotted for left and right hemisphere separately, from the group-level cluster maps for Controls, LTLE patients, and RTLE patients (from top to bottom).

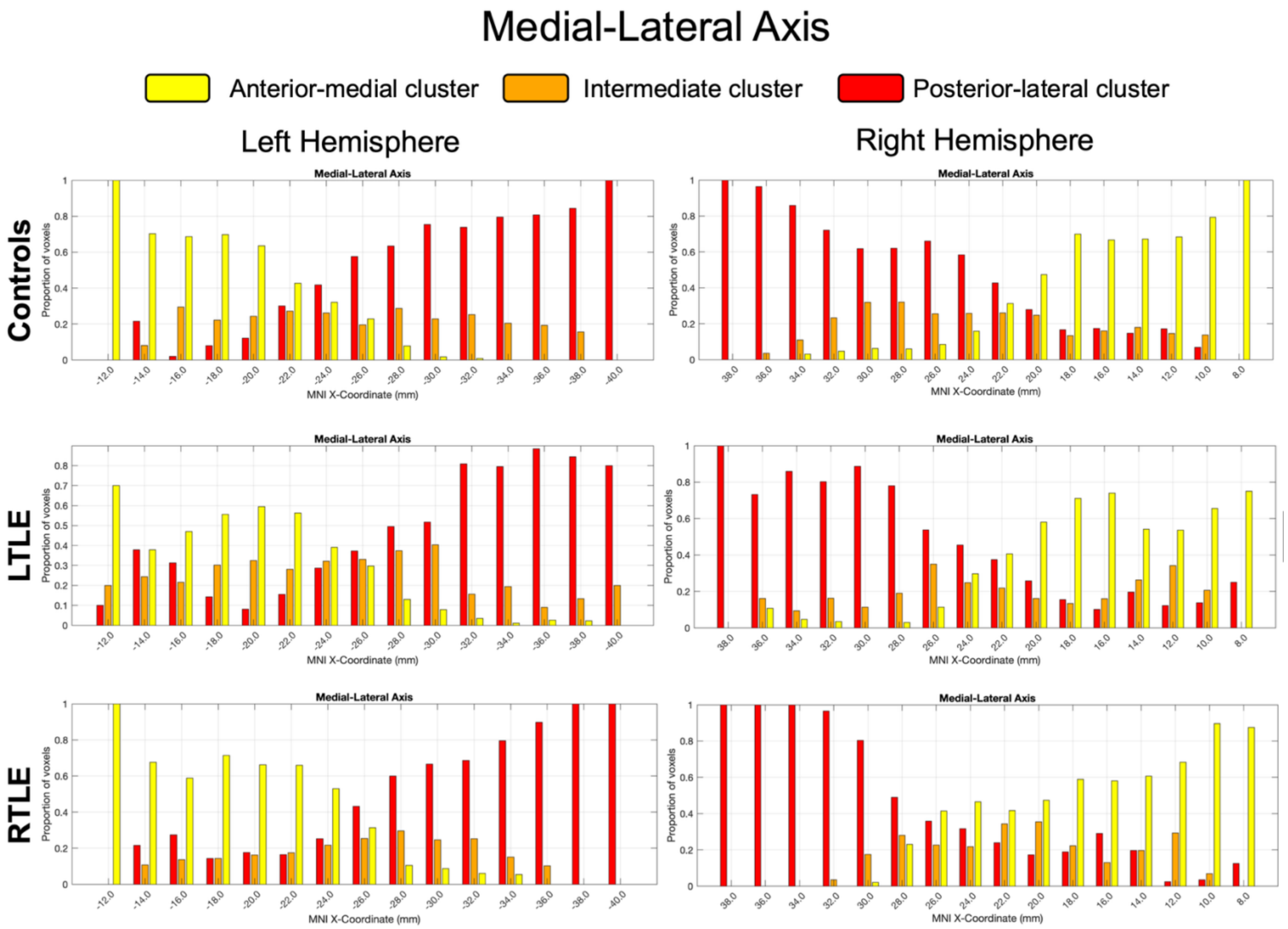

Figure S6. Spatial distribution of the autocorrelation clusters along the medial-lateral axis. The proportion of voxels assigned to the anterior-medial cluster, intermediate cluster, and posterior-lateral cluster across 2 mm slices in MNI space along the medial-lateral axis (X direction). The spatial distribution of autocorrelation clusters is plotted for left and right hemisphere separately, from the group-level cluster maps for Controls, LTLE patients, and RTLE patients (from top to bottom).
